# Clinical Presentation and Etiology of Thrombocytopenia Among Patients at a Tertiary Care Hospital in the Kingdom of Saudi Arabia

**DOI:** 10.7759/cureus.107700

**Published:** 2026-04-25

**Authors:** Muhammad Farooq, Mohammad A Zafer, Mohammed AlWazna, Ahmed S Alrashah, Sarah Alkhamis, Saleh K Alsarem, Lina Aldakhil

**Affiliations:** 1 Internal Medicine, King Saud Medical City, Riyadh, SAU; 2 Pharmacy, Al Maarefa University, Riyadh, SAU; 3 Hematology, King Fahad Medical City, Riyadh, SAU; 4 Research Center, King Saud Medical City, Riyadh, SAU

**Keywords:** bleeding, kingdom of saudi arabia, king saud medical city, malaria, petechiae, riyadh first health care cluster, thrombocytopenia

## Abstract

Background

Thrombocytopenia is a common hematological disorder encountered in clinical practice. To improve diagnostic efficiency and patient outcomes, it is important to understand its frequent clinical manifestations and predominant etiologies in different healthcare settings. This study aimed to describe the typical clinical presentations and underlying causes of thrombocytopenia among patients at a tertiary care hospital in the Kingdom of Saudi Arabia.

Study design

A single-center, retrospective cross-sectional study.

Materials and methods

Patients with thrombocytopenia (platelet count < 150,000/microL) admitted between January and December 2022 were included. Patients with pseudothrombocytopenia were excluded. Demographic, clinical, and laboratory data were collected through manual review of electronic medical records.

Etiologies were recorded, and statistical analysis was performed using Stata software. Here and throughout, n denotes the number of patients contributing data to a given comparison, and N denotes the total cohort (N = 114). Where applicable, denominators were adjusted for missing data.

Results

Of 114 patients, 73 (64.04%) were male. The mean age was 40.80 ± 15.93 years, and the mean length of hospital stay was 11.83 ± 15.73 days. The mean platelet count was 51,480 ± 39,690/microL. Mild thrombocytopenia was observed in 19 (16.67%) patients, moderate in 31 (27.19%), and severe in 64 (54.14%). Infection was the most common etiology (n = 48, 42.11%), with *Plasmodium* species accounting for the largest number of cases (n = 25). Bleeding manifestations were observed in 72 (63.07%) patients, most commonly epistaxis (n = 16, 14.04%), ecchymosis (n = 13, 11.4%), and petechiae (n = 9, 7.89%). Patients with bleeding, particularly petechiae, ecchymosis, epistaxis, and gum bleeding, had significantly lower platelet counts than those without bleeding symptoms.

Conclusion

Infections were the predominant cause of thrombocytopenia in this cohort. Lower platelet counts were significantly associated with bleeding manifestations, particularly epistaxis, emphasizing the need for early recognition and appropriate clinical management.

## Introduction

Platelets are small, enucleated cells that circulate in the blood and play a significant role in hemostasis. The normal platelet count is 150,000-450,000/microL of blood [1]. Thrombocytopenia is defined as a platelet count < 150,000/microL of blood [1]. The risk of pseudothrombocytopenia (i.e., falsely low platelet count) should be ruled out before performing any further evaluation. Pseudothrombocytopenia can occur in several settings and can be identified by reviewing the peripheral blood smear and/or repeating a complete blood count (CBC) using a non-ethylenediaminetetraacetic acid (EDTA) anticoagulant. Incompletely mixed or inadequately anticoagulated samples may form a clot that traps platelets in the collecting tube and prevents them from being counted [2].

Thrombocytopenia is commonly encountered among patients presenting to emergency departments and among those admitted to intensive care units. It has been reported in approximately 20% of medical patients and in one-third of surgical ICU patients, where it may represent an important abnormal laboratory finding associated with bleeding risk and adverse clinical outcomes [3]. It can be classified into three main categories according to severity. Mild thrombocytopenia is defined as a platelet count of 100,000-150,000/microL of blood. Moderate thrombocytopenia is defined as a platelet count of 50,000-100,000/microL of blood. Severe thrombocytopenia is defined as a platelet count of < 50,000/microL of blood [4]. However, this categorization must be considered along with the type of illness. This is because some conditions are associated with an elevated risk of bleeding (a platelet count of < 50,000/microL), and other illnesses may not be related to an increased risk until the platelet count reaches < 10,000/microL. Therefore, bleeding manifestations vary per case, and they cannot be predicted in certain circumstances [5].

In several cases, patients with thrombocytopenia remain asymptomatic, and they are diagnosed upon routine CBC. Some individuals with thrombocytopenia may experience external bleeding symptoms, such as nosebleeds and gum bleeding. Others may develop hematuria or gastrointestinal bleeding [6].

Meanwhile, women with thrombocytopenia may experience heavier or longer menstrual cycles and/or breakthrough menstrual bleeding. Bruising, particularly purpura on the forearms and petechiae on the feet, legs, and mucous membranes, may result from spontaneous bleeding under the skin. Some clinical signs, such as purpura and hematuria, can be clinical predictors, and life-threatening bleeding can be predicted and expected if purpura and/or hematuria are the presenting symptoms [6]. Thrombocytopenia can be associated with thrombosis, as observed in conditions such as disseminated intravascular coagulopathy, thrombotic thrombocytopenic purpura, antiphospholipid antibody syndrome, and heparin-induced thrombocytopenia. Thus, clinicians should consider the underlying etiologies when managing patients with thrombocytopenia [7].

Several pathophysiological mechanisms are associated with the development of thrombocytopenia. The most basic mechanisms are decreased platelet production by the bone marrow or increased platelet destruction by the immune system and/or the reticuloendothelial system.

Thrombocytopenia has several etiologies. Infection is one of the major precipitants of this condition and, in some cases, may act in combination with other factors. Common infectious causes of thrombocytopenia include viral infections such as dengue, Epstein-Barr virus, hepatitis B, and hepatitis C; parasitic infections such as malaria; and bacterial infections that may progress to sepsis. Certain medications are also implicated in thrombocytopenia. Valproic acid has been associated with both dose-dependent bone marrow suppression and immune-mediated thrombocytopenia. Linezolid has also been associated with thrombocytopenia, possibly through bone marrow suppression related to mitochondrial toxicity, including impairment of cytochrome c oxidase function [8-12].

Other medications may lead to bone marrow suppression or direct toxic effects that can cause thrombocytopenia [8-12]. Malignancy, via either bone marrow infiltration by malignant cells or the direct toxic effects of chemotherapeutic agents or radiotherapy, is an important etiology of thrombocytopenia. Primary bone marrow disorders such as aplastic anemia, leukemia, and lymphoma may present with thrombocytopenia [12]. Immune-mediated thrombocytopenia is another important category of thrombocytopenia. It is divided into idiopathic thrombocytopenic purpura and autoimmune disease-mediated thrombocytopenia, such as systemic lupus erythematosus and rheumatoid arthritis [11]. Increased platelet destruction via hypersplenism with or without splenomegaly may be another cause of thrombocytopenia [13]. Furthermore, in certain cases, the etiology behind the development of thrombocytopenia remains unknown. These cases are classified as thrombocytopenia of undetermined cause [14].

This study primarily aimed to describe the most common etiologies of thrombocytopenia among patients at a tertiary care center in the Kingdom of Saudi Arabia and to evaluate the association between thrombocytopenia severity and selected clinical manifestations and complications. It secondarily aimed to examine the association between thrombocytopenia severity and length of hospital stay.

## Materials and methods

Study design and participants

This retrospective, cross-sectional study was conducted at King Saud Medical City in Riyadh from January 1, 2022, to December 31, 2022.

Ethical committee approval

Ethical committee approval was obtained prior to initiating the study. The need for informed consent was waived by the committee due to the study’s design. IRB approval details are in Appendix D.

Inclusion criteria

The inclusion criteria were patients aged ≥ 18 years who were admitted to the hospital from January 1, 2022, to December 31, 2022, and who had platelet counts of < 150,000/microL.

Exclusion criteria

Patients were excluded if they were pregnant or lactating, had evidence of pseudothrombocytopenia, or were taking antiplatelet medications such as aspirin or clopidogrel. Pseudothrombocytopenia is an in-vitro specimen-collection phenomenon that may occur when an anticoagulant used for testing a blood specimen causes unintentional platelet clumping, resulting in a spuriously low platelet count. It occurs most often in EDTA-anticoagulated blood samples; however, other anticoagulants such as citrate, heparin, and oxalate have also been implicated [2,14].

Definitions and study variables

Thrombocytopenia severity was classified as mild, moderate, or severe (Appendix A). Mild thrombocytopenia was defined as a platelet count of 100,000-150,000/microL, moderate thrombocytopenia as 50,000-100,000/microL, and severe thrombocytopenia as < 50,000/microL. The length of hospital stay was calculated from the day of admission until the day of discharge or death.

Data collection and procedures

The principal investigator screened patients admitted during the study period who had primary diagnoses of thrombocytopenia, bicytopenia, or pancytopenia. Patients who met the inclusion criteria were included in the study; the ICD-10 codes used for case identification are provided in Appendix C. The list of selected patients was handed over to co-investigators for data collection (Appendix B). The data collection process utilized the local electronic medical record system, Hospital Information System (HIS) (eSiHi, Ministry of Health, Riyadh, Saudi Arabia), in addition to physical patient medical record files provided by the Medical Records Department of King Saud Medical City. A custom spreadsheet was created using Google Sheets for data recording. It listed data on demographic characteristics such as age, sex, and ethnicity; clinical manifestations such as bleeding from a specific site, petechiae, and purpura; symptoms of anemia including shortness of breath, fatigue, and palpitations; laboratory findings such as hemoglobin levels, platelet counts, liver function test results, coagulation profiles, kidney function test findings, bone marrow aspiration and biopsy results; and the final diagnosis. Data validation was independently performed by different co-investigators. After the data collection, all data were exported into a Microsoft Excel (Redmond, USA) sheet, which was utilized as the data source for statistical analysis.

Here and throughout, n denotes the number of patients contributing data to the specified comparison, and N denotes the total cohort (N = 114). Where data for a given variable were missing, the analysis denominator is reported explicitly for that comparison.

Statistical analysis

Data were analyzed using Stata version 15 (StataCorp LLC, College Station, TX, USA). Etiologies, including infection, vitamin B12 deficiency, and hypersplenism, were summarized as frequencies and percentages. Because platelet counts were not normally distributed, Spearman’s rank correlation was used to assess the association between platelet count and length of hospital stay, age, and white blood cell (WBC) count. Correlations between platelet count and continuous variables (length of stay, age, WBC, hemoglobin, mean corpuscular volume [MCV]) were evaluated using Spearman’s rank correlation on a complete-case basis (n = 110).

Differences in platelet counts between patients with and without bleeding manifestations were assessed using the Wilcoxon rank-sum test. The association between disease categories and the degree of thrombocytopenia (mild, moderate, and severe) was examined using the chi-square test.

Finally, linear regression analysis was performed to identify independent disease factors and bleeding manifestations associated with platelet count. A p-value < 0.05 was considered statistically significant.

## Results

Of 114 patients, 73 (64.04%) were male. There was no statistically significant difference between male and female patients in terms of thrombocytopenia severity (p = 0.19). The mean age of the participants was 40.80 ± 15.93 years (range: 19-90), with a median age of 38 years (IQR: 28-48). The mean length of hospital stay was 11.83 ± 15.73 days, and the median length of hospital stay was six days (IQR: 4-12). The demographic and clinical characteristics of the study population are summarized in Table 1. There was no statistically significant difference in disposition (discharged, deceased, or transferred) according to thrombocytopenia severity (p = 0.19). Platelet count was negatively correlated with length of hospital stay, but this association was not statistically significant (p = 0.327). The mean platelet count was 51,480 ± 39,690/microL, with a median of 47,000/microL (IQR: 63,000/microL; range: 2,000-146,000/microL). Mild thrombocytopenia was observed in 19 (16.67%) patients, moderate in 31 (27.19%), and severe in 64 (54.14%). Infection was the most common etiology of thrombocytopenia (n = 48 [42.11%]), with malaria accounting for the highest number of cases (n = 25). Table 2 shows the other causes of thrombocytopenia.

**Table 1:** Demographic and clinical characteristics of the study population (N = 114) Data are presented as mean ± standard deviation, median (interquartile range), or number (%), as appropriate. IQR: Interquartile range, SD: Standard deviation

Variable	Value
Age, years (mean ± SD)	40.80 ± 15.93
Age, years (median [IQR])	38 (28–48)
Length of hospital stay, days (median [IQR])	6.0 (4.0–12.0)
Gender	
Male	73 (64.04%)
Female	41 (35.96%)
Disposition	
Discharged home	92 (83.6%)
Discharged against medical advice (DAMA)	5 (4.5%)
Expired	12 (10.9%)
Transferred	1 (0.9%)

**Table 2:** Etiologies of thrombocytopenia

Etiology	n (%)
Infection	48 (42.11%)
Autoimmune disease	23 (20.13%)
Hematological malignancies	19 (16.67%)
Undetermined causes	7 (6.14%)
Vitamin B12 deficiency	6 (5.26%)
Hypersplenism	5 (4.39%)
Drug-induced thrombocytopenia	3 (2.63%)
Non-hematological malignancies	3 (2.63%)

Moreover, there was no significant correlation between platelet count and age, WBC count, hemoglobin level, or mean corpuscular volume (Table 3). In addition, as shown in Table 4, the etiologies of thrombocytopenia, including infection, drug-induced causes, malignancy, and autoimmunity, did not differ significantly across thrombocytopenia severity categories.

**Table 3:** Spearman's rank correlation between platelet count and length of hospital stay, age, WBC count, hemoglobin level, and MCV Analysis limited to patients with complete data for all variables (n = 110); four patients had missing data for one or more variables. MCV: mean corpuscular volume, WBC: White blood cells

Variable	n	Coefficient	p-value
Length of hospital stay	110	−0.0943	0.3271
Age	110	0.0106	0.9124
WBC count	110	−0.0567	0.5561
Hemoglobin Level	110	−0.1794	0.0608
MCV	110	0.1137	0.2368

**Table 4:** Comparison of etiologies and clinical features across thrombocytopenia severity categories AML: Acute myeloid leukemia, APL: Acute promyelocytic leukemia, ALL: Acute lymphoblastic leukemia, MDS: Myelodysplastic syndromes, SLE: Systemic lupus erythematosus

Variables	Mild thrombocytopenia	Moderate thrombocytopenia	Severe thrombocytopenia	Total number of patients	Chi-square	p-value
Plasmodium Vivax- Yes	3 (15.79)	5 (16.13)	6 (9.38)	14 (12.28)	1.1449	0.564
Plasmodium Falciparum- Yes	2 (10.53)	6 (19.35)	3 (4.69)	11 (9.65)	5.1736	0.075
Viral infection- Yes	4 (21.05)	6 (19.35)	11 (17.19)	21 (18.42)	0.1703	0.918
TB- Yes	0 (0)	1 (3.23)	3 (4.69)	4 (3.51)	0.9609	0.619
Aplastic- Yes	1 (5.26)	0 (0)	5 (7.81)	6 (5.26)	2.5564	0.279
Lymphoma- Yes	1(5.26)	1 (3.23)	3 (4.69)	5 (4.39)	0.1482	0.929
AML- Yes	0 (0)	0 (0)	4 (6.25)	4 (3.51)	3.2386	0.198
APL- Yes	0 (0)	0 (0)	2 (3.12)	2 (1.75)	1.5904	0.451
ALL- Yes	0 (0)	0 (0)	1 (1.56)	1 (0.88)	0.7882	0.674
MDS- Yes	0 (0)	0 (0)	2 (3.12)	2 (1.75)	1.5904	0.451
SLE- Yes	3 (15.79)	1 (3.23)	2 (3.12)	6 (5.26)	5.0671	0.079
Autoimmune diseases- Yes	2 (10.53)	4 (12.9)	2 (3.12)	8 (7.02)	3.4904	0.175
Vitamin B12 deficiency- Yes	1 (5.26)	3 (9.68)	2 (3.12)	6 (5.26)	1.7983	0.407
Hypersplenism- Yes	0 (0)	2 (6.45)	3 (4.69)	5 (4.39)	1.2009	0.549
Drug-induced thrombocytopenia- Yes	0 (0)	0 (0)	3 (4.69)	3 (2.63)	2.4071	0.3
Lung cancer- Yes	0 (0)	0 (0)	1 (1.56)	1 (0.88)	0.7882	0.674
Lymphoma- Yes	1 (5.26)	3 (9.68)	4 (6.25)	8 (7.02)	0.4835	0.785
Hepatomegaly- Yes	1 (5.26)	4 (12.9)	5 (7.81)	10 (8.77)	1.0271	0.598
Splenomegaly Yes	2(10.53)	5 (16.13)	10 (15.62)	17 (14.91)	0.3498	0.84
Fever- Yes	7 (36.84)	16 (51.61)	27 (42.19)	50 (43.86)	1.2095	0.546
Thrombosis- Yes	0 (0)	1 (3.23)	0 (0)	1 (0.88)	2.7011	0.259
CNS hemorrhage- Yes	0 (0)	0 (0)	2 (3.12)	2 (1.75)	1.5904	0.451

Bleeding manifestations were observed in 63.07% of patients. Among them, epistaxis (14.04%) was the most common, followed by ecchymosis (11.4%) and petechiae (7.89%). Other clinical manifestations included fever (48.86%), hepatomegaly (8.77%), and splenomegaly (14.91%).

Patients with bleeding manifestations had lower platelet counts: petechiae, 9 × 10^9/L vs 49 × 10^9/L (p < 0.001); ecchymosis, 15 × 10^9/L vs 48 × 10^9/L (p = 0.04); epistaxis, 10.5 × 10^9/L vs 50.5 × 10^9/L (p < 0.001); and gum bleeding, 4 × 10^9/L vs 48 × 10^9/L (p < 0.001) (Table 5).

**Table 5:** Wilcoxon rank-sum test assessing platelet counts by bleeding manifestations (available-case analysis) Analysis set n = 76 for bleeding comparisons (available platelet counts); counts for “with” and “without” refer to this analysis subset.

Manifestation	With Median (25th quartile-75th quartile), ×10^9/L	Without Median (25th quartile-75th quartile), ×10^9/L	Wilcoxon rank-sum (z-value)	p-value
Petechiae	9 (4-15)	49 (20-85)	-3.42	<0.001
Ecchymosis	15 (4-41)	48 (20-81)	-2.04	0.04
Epistaxis	10.5 (4-22)	50.5 (21-89)	-4.25	<0.001
Gum bleeding	4 (4-12)	48 (20-85)	-3.49	<0.001

Finally, malignancy, drug-induced thrombocytopenia, hemoglobin level, epistaxis, and gum bleeding were negatively associated with the platelet count (Table 6).

**Table 6:** Multiple linear regression analysis of the association between platelet count (×10^9/L) and malignancy, drug-induced thrombocytopenia, hemoglobin level (g/dL), epistaxis, and gum bleeding

Predictor	Coef	SE	p-value	(95% confidence interval)
Malignancy – No (reference)	Reference	Reference	Reference	Reference
Malignancy – Yes	−28.12776	8.416861	0.001	(−44.81 to −11.44)
Drug-induced thrombocytopenia – No (reference)	Reference	Reference	Reference	Reference
Drug-induced thrombocytopenia – Yes	−55.09839	19.92312	0.007	(−94.58 to −15.60)
Hemoglobin level (g/dL)	−2.451448	0.8647852	0.005	(−4.165 to −0.737)
Epistaxis – No (reference)	Reference	Reference	Reference	Reference
Epistaxis – Yes	−31.14087	9.560825	0.002	(−50.09 to −12.18)
Gum bleeding – No (reference)	Reference	Reference	Reference	Reference
Gum bleeding – Yes	−26.52539	12.49948	0.036	(−51.30 to −1.74)

## Discussion

Thrombocytopenia is a frequently encountered hematological abnormality in hospital practice and may arise from a broad spectrum of underlying etiologies. In the present study, males comprised 64.04% of the cohort, resulting in a male-to-female ratio of 1.7:1. This pattern is consistent with several prior studies that likewise reported a male predominance among patients with thrombocytopenia [15-20].

The mean age of our participants was 40.80 ± 15.93 years, with a median age of 38 years. Previous studies have shown some variability in the age distribution of thrombocytopenia; however, many reports place the predominant age range in early to middle adulthood, which is consistent with our findings [12,15-22]. Although age was positively associated with platelet count in our cohort, this association was not statistically significant. Likewise, thrombocytopenia severity did not differ significantly with respect to age.

The mean platelet count in our study was 51,480 ± 39,690/microL, with a median of 47,000/microL (IQR: 63,000/microL; range: 2,000-146,000/microL). Mild thrombocytopenia was observed in 19 patients (16.67%), moderate in 31 (27.19%), and severe in 64 (54.14%). In contrast to our findings, several previous studies have reported mild or moderate thrombocytopenia as the predominant severity category, with a lower proportion of severe thrombocytopenia than that observed in our cohort [16,17,22,23]. This difference may be explained by the tertiary care setting of our study, which is more likely to receive referrals for severe or complicated cases, whereas milder cases may be managed in primary or secondary care settings.

Bleeding manifestations were observed in 63.07% of the study population, with epistaxis (14.04%) being the most common, followed by ecchymosis (11.4%) and petechiae (7.89%). Other clinical manifestations included fever (48.86%), hepatomegaly (8.77%), and splenomegaly (14.91%). Previous studies have similarly shown that mucocutaneous bleeding manifestations, particularly petechiae, ecchymosis, epistaxis, and gum bleeding, are common clinical presentations of thrombocytopenia, although the reported frequencies vary across cohorts [12,16,19,21,24-27]. In our study, lower platelet counts were significantly associated with petechiae, ecchymosis, epistaxis, and gum bleeding. These findings support the clinical relevance of mucocutaneous bleeding as a marker of more severe thrombocytopenia.

No statistically significant correlation was found between platelet count and length of hospital stay, age, white blood cell count, hemoglobin level, or mean corpuscular volume. In addition, no significant associations were identified between thrombocytopenia severity and the major etiologic categories or clinical biomarkers evaluated in our analysis. These findings suggest that, within our cohort, bleeding manifestations were more closely associated with lower platelet counts than were the broader disease categories themselves.

Infection was the most common etiology of thrombocytopenia in our study, accounting for 42.11% of cases, with malaria representing the single largest contributor. Autoimmune diseases and hematological malignancies were the next most frequent etiologies. Several previous studies have also identified infectious causes as the predominant contributors to thrombocytopenia, with dengue, malaria, and sepsis among the most commonly reported etiologies in different settings [15,16,19,21-23,28]. Thus, our findings are consistent with the broader literature and reinforce the importance of considering infectious causes early in the evaluation of thrombocytopenia, particularly in regions where such conditions remain prevalent.

Malaria appears to be especially important in our cohort. This may be explained by its well-recognized pathogenic mechanisms, including immune-mediated platelet destruction, splenic sequestration, and impaired thrombopoiesis. In contrast, some previously reported cohorts have found megaloblastic anemia, chronic liver disease, aplastic anemia, or other noninfectious causes to be more prominent [12,16,22]. Such differences likely reflect geographic, dietary, epidemiologic, and healthcare-setting variation across study populations.

Vitamin B12 deficiency was identified in only 5.26% of our cohort. This relatively low frequency may reflect regional differences in diet and disease burden compared with studies from other populations in which megaloblastic anemia was more common [12]. Similarly, while malignancy and drug-induced thrombocytopenia were less frequent etiologies overall, both remained clinically important in the regression analysis because they were negatively associated with platelet count.

The observed associations of malignancy, drug-induced thrombocytopenia, low hemoglobin level, epistaxis, and gum bleeding with lower platelet counts are clinically meaningful. These findings emphasize the importance of considering both underlying pathology and presenting bleeding manifestations when evaluating patients with thrombocytopenia. However, given the retrospective cross-sectional design of the study, these findings should be interpreted as associations rather than causal relationships.

This study has several limitations. First, its retrospective design limits causal inference and depends on the accuracy and completeness of the medical records reviewed. Second, the study was conducted at a large tertiary care center, which may have introduced referral bias and may limit generalizability to the broader community. Third, as a single-center study, the sample size was relatively limited. Despite these limitations, the study provides useful regional data on the clinical presentation and etiological spectrum of thrombocytopenia and supports the observation that infectious causes remain predominant in this setting. Future multicenter prospective studies are needed to validate these findings and to better define the relative contributions of different etiologies across diverse healthcare settings.

Scientific nomenclature

In this manuscript, scientific nomenclature was used in accordance with CDC guidelines. Genus and species names of microorganisms (e.g., *Plasmodium falciparum* and *Plasmodium vivax*) were italicized throughout the text, except in the title, headings, tables, and figures, where italics were intentionally omitted according to journal guidelines. Species names were consistently written in lowercase, whereas genus names were capitalized. Abbreviations of species names were correctly formatted (e.g., *P. falciparum*). No genes or proteins requiring specific nomenclature were referenced in this manuscript.

## Conclusions

Thrombocytopenia has a broad spectrum of etiologies, with infection being among the most common. Given its potential for serious complications, prompt recognition and thorough clinical evaluation are essential to identify the underlying cause. Clinicians should consider context-specific common etiologies and be aware that symptoms such as epistaxis and gum bleeding may serve as early indicators of clinically significant thrombocytopenia. Although this study was retrospective, it highlighted important aspects of thrombocytopenia presentation and evaluation and underscored the need for further research using more robust study designs.

## Appendices

Appendix A 

Case Definition of Thrombocytopenia

Thrombocytopenia was defined as a platelet count of less than 150,000/microL. Severity was categorized as:

Mild: 100,000-149,000/microL Moderate: 50,000-99,000/microL Severe: < 50,000/microL

Appendix B 

Data Collection Template

A standardized data abstraction form was used to collect the following variables from medical records:

Patient demographics (age, sex) Presenting symptoms (e.g., bleeding, fatigue) Platelet count and CBC parameters Final diagnosis Length of hospital stay Clinical outcomes (discharged, transferred, or deceased)

Appendix C 

ICD-10 Codes Used for Patient Identification

Patients were identified using ICD-10 codes relevant to thrombocytopenia and related etiologies, including:

D69.6: Thrombocytopenia, unspecified D69.3: Idiopathic thrombocytopenic purpura D69.5: Secondary thrombocytopenia A41.x: Sepsis B50-B54: Malaria C00-C97: Malignancies

Appendix D 

Institutional Review Board (IRB) Approval

This study received approval from the Institutional Review Board (IRB) at King Saud Medical City.

Proposal Title: Clinical Presentation and Etiology of Thrombocytopenia at a Tertiary Care Hospital IRB Reference Number: H1RI-04-Jan23-01 Category of Approval: Exempt Approval Validity: January 9, 2023 - January 8, 2024 Principal Investigator: Dr. Mohammad Ahmed Saad Al Gahtani

Acknowledgement

We acknowledge the medical record department and IT department for their support in collecting data.

Conflict of interest

The authors declare no conflicts of interest.
